# Insulin-Loaded Chitosan–Cellulose-Derivative Hydrogels: In Vitro Permeation of Hormone through Strat-M^®^ Membrane and Rheological and Textural Analysis

**DOI:** 10.3390/polym16182619

**Published:** 2024-09-16

**Authors:** Aneta Ostróżka-Cieślik, Claire Strasser, Barbara Dolińska

**Affiliations:** 1 Department of Pharmaceutical Technology, Faculty of Pharmaceutical Sciences in Sosnowiec, Medical University of Silesia, Jedności Street 10, 41-200 Sosnowiec, Poland; bdolinska@sum.edu.pl; 2 NETZSCH-Geratebau GmbH, Wittelsbacherstraße 42, 95100 Selb, Germany; claire.strasser@netzsch.com

**Keywords:** hydrogels, insulin, cellulose ethers, chitosan, rheology, texture, Strat-M^®^ membrana

## Abstract

This work is part of the current research trend to develop a hydrogel carrier of insulin to promote wound healing. Topically applied insulin promotes keratinocyte proliferation and migration, increases collagen synthesis, reduces inflammation and oxidative stress, and exhibits antimicrobial activity. The aim of this study was to design an insulin hydrogel matrix based on selected cellulose derivatives (methylcellulose, hydroxyethylcellulose, and hydroxypropylmethylcellulose) and chitosan. Rheological parameters of the formulations were evaluated using rotational rheometry and an oscillation test. Textural tests were performed. In vitro pharmaceutical insulin availability studies were carried out using the innovative Strat-M^®^ membrane to imitate the skin barrier. It was found that the pharmaceutical formulation of insulin based on chitosan and methylcellulose showed an acceptable balance between rheological and textural parameters and ease of application. The API was released from the carrier in a prolonged manner, eliminating the need to apply the formulation several times per day. The developed hydrogel shows potential for use in clinical practice.

## 1. Introduction

Despite major medical advances, chronic wound management is a global health, economic, and epidemiological problem. In recent years, the number of patients diagnosed with chronic wounds has been increasing, with a deterioration in their quality of life due to the need for continuous local treatment and the accompanying pain, odor, and even immobilization. It is estimated that 1–2% of the population of developed countries struggle with chronic wound management [1]. This problem will be exacerbated by an aging population. The definition states that a chronic wound is a localized skin lesion caused by disease or trauma, with a high risk of infection, that does not heal within 6–8 weeks [2,3]. The most common causes are venous leg ulcers, ischemic leg wounds, mixed wounds of venous and non-ischemic etiology, diabetic foot syndrome, and decubitus ulcers [4,5]. Chronic venous disease affects 40% of the world’s population, among which venous ulcers occur in 1–2% of patients. The risk of venous ulcers increases by approximately 4% in patients over 65 years of age [6]. In turn, the International Diabetes Federation (IDF) have reported that 537 million people aged 20–79 years had diabetes worldwide in 2021 [7], while 18.6 million patients were diagnosed with the diabetes complication of diabetic foot [5]. Decubitus ulcers, on the other hand, are a health problem for more than 0.85 million patients worldwide and are estimated to be increasing among patients over the age of 95 years [8]. Given the high proportion of patients struggling with chronic wounds, it is important to develop new preventive and therapeutic technologies to limit the progression of the disease and reduce the economic burden on patients and health systems.

There is currently a great deal of research into the development of insulin (INS) carriers for topical therapy of chronic wounds, particularly in patients with diabetic foot ulcers. Insulin exhibits a multidirectional effect to stimulate wound healing. It promotes keratinocyte proliferation and migration, increases collagen synthesis, reduces inflammation and oxidative stress, and exhibits antimicrobial activity. Liu et al. [9] suggest that insulin can promote keratinocyte migration and proliferation (dose-dependently) through the (IR)/PI3K/Akt receptor pathway, affecting increased integrin α3 expression and LN332 (Laminin 332) deposition. Through interaction with the IGF-1 receptor, the hormone induces the formation of collagen [10], an important component of the extracellular matrix (ECM). Collagen in both fibrous and solubilized forms is involved in regulating the phases of wound healing [11]. The migration of keratinocytes and increased collagen production influence the formation of a new epithelial layer on the wound surface [12]. Stimulation of insulin receptors induces proangiogenic effects of the hormone. INS regulates endothelial cell migration, proliferation, and formation, increases vascular endothelial growth factor (VEGF) expression, and decreases anti-angiogenic protein expression [13]. It also exhibits anti-inflammatory effects by modulating inflammation. It reduces the activation of the transcription factor nuclear factor kappa B (NF-κB) and the expression of pro-inflammatory cytokines such as tumor necrosis factor alpha (TNF-α) through the regulation of NF-kβ^P50/P50^ [14,15,16]. It also induces the expression of anti-inflammatory cytokines, i.e., interleukin 6 (IL-6) and interleukin 10 (IL-10), through activation of STAT6 (signal transducer and activator of transcription 6), STAT3 (signal transducer and activator of transcription 3), and transcription factor c-Maf [16]. Insulin increases catalase (CAT) and reduces glutathione (GSH) and vitamin E concentrations [17]. It also up-regulates Nrf2 (nuclear factor erythroid 2-related factor)-dependent antioxidant enzymes [18], indicating its antioxidant potential. Insulin can also induce phagocytic activity of neutrophils showing antimicrobial activity [19].

Insulin is a peptide hormone for which the development of technology to prepare an effective carrier that will give it stability and therapeutic activity is a challenge. The hormone is sensitive to enzymatic degradation at the wound site and changes in pH and temperature. A novel hydrogel formulation may be the optimal delivery system for INS [20]. The hydrogel matrix can act as a physical barrier between the external environment and the wound, effectively protecting the affected area from external damage. An optimal hydrogel should absorb exudate in exuding wounds, moisturize dry wounds by releasing the moisture contained in the matrix, stop bleeding, and facilitate dressing changes. It has been found that therapeutic protein molecules can be effectively incorporated into the hydrogel matrix, effectively protecting them from proteolytic and chemical degradation and ensuring their prolonged release [21,22]. The advantage of hydrogel formulations is the relatively simple technology for their preparation from synthetic, natural, and modified polymers.

The present work is a continuation of our research towards the development of an insulin hydrogel formulation [22,23]. Baseline studies of the hydrogel matrix obtained by cross-linking chitosan (CS) with cellulose derivatives (methylcellulose/ME, hydroxyethylcellulose/HEC, hydroxypropylmethylcellulose/HPMC) in hybrid systems (CS/MC, CS/HEC, CS/HPMC) as potential carriers of insulin were carried out. Chitosan is a natural polysaccharide obtained by deacetylation of chitin, most commonly derived from arthropod carapaces, insect exoskeletons, or fungal cell walls. The unique properties of chitosan, such as anti-inflammatory, antimicrobial, antioxidant, and hemostatic effects, make it conducive to skin regeneration and preferred for the preparation of dermatological hydrogels. It is biocompatible, biodegradable, and non-toxic, it exhibits low immunogenicity, and the technology to obtain it is relatively inexpensive. Its antioxidant activity is due to the presence of amino and hydroxyl groups in its structure, which can bind oxygen free radicals [24]. It can exert anti-inflammatory effects by stimulating phagocytes and inducing NK (natural killer) cells [25]. Chitosan has an effect on stopping bleeding, via promoting blood clotting and fibrinolysis [26]. It shows antimicrobial capacity against Gram-negative and Gram-positive bacteria, e.g., *S. epidermidis*, *S. mutans*, *S. aureus*, *P. aeruginosa*, *E. coli*, *E. faecium*, *S. typhimurium*, *M. flavus*. Due to its cationic nature, it interacts electrostatically with negatively charged microbial cell membranes [27,28]. In addition, it shows the ability to penetrate bacterial cell membranes, interfering with the transcription and translation of their genetic material [29]. Yan et al. [30] found that the chitosan–gentamicin conjugate was able to affect total protein synthesis in granulation tissue by increasing hydroxyproline content. This facilitated collagen fibrogenesis, minimized the expression of pro-inflammatory cytokines, and accelerated wound healing. In turn, other studies have confirmed that chitosan promotes the synthesis of TGF-beta1 (transforming growth factor-beta 1) and PDGF (platelet-derived growth factor), which stimulate fibroblast proliferation and influence the production of extracellular matrix (ECM) components in the later stages of wound healing [31]. Smith et al. [32] suggested that chitosan may increase epithelial permeability by disrupting intercellular junctions, potentially improving the permeation of APIs through the skin. Its disadvantages are its low water solubility and the need for solvents with an acidic pH, which limits its use in medicine [33]. It also has low mechanical strength, which can be improved by blending it with other natural or synthetic polymers. Semi-synthetic cellulose derivatives (MC, HEC, HPMC), widely used in pharmaceutical formulations, can be effective in this respect. They are distinguished by their optimal mechanical strength, high stability, biocompatibility, and transparency, which allows the wound bed to be observed [34].

It has been suggested that the chitosan and cellulose contained in most biomass materials have very good biochemical properties and high potential in the development of pharmaceutical and biotechnology industries [35,36]. Kumar et al. [37] developed nanocomposite hydrogel sprayers based on polyvinyl alcohol/chitosan with AgNPs (PVA/CH/Ag) for skin application. The developed dressing showed a concentration-dependent antimicrobial effect (against *S. aureus* and *E. coli*). In another study, a pH-sensitive, self-healing taurine hydrogel based on carboxymethyl chitosan and oxidized hyaluronic acid (CMCS/OHA/Tau) was designed. The preparation effectively promoted diabetic wound healing in a rat model. The formulation was observed to be highly biocompatible with the wound environment and was found to inhibit inflammatory cytokine synthesis and cell migration [38]. Cai et al. [39] prepared a sprayable hydrogel whose matrix consisted of three polymers: chitosan, sodium carboxymethyl cellulose, and sodium alginate. The dressing had antimicrobial, anti-inflammatory, and wound healing-promoting properties. Increased skin cell proliferation and inhibition of apoptosis were observed. The research conducted towards the development of effective hydrogel wound dressings indicates that this is a timely and important topic.

The aim of this study was to develop a hybrid carrier for insulin based on chitosan and cellulose derivatives, for the purpose of making a prescription drug. To our knowledge, comparative studies within the proposed groups of polysaccharide hydrogels with chitosan and insulin have not yet been conducted.

## 2. Materials and Methods

### 2.1. Materials

Insulin Insulatard Penfil (INS, human insulin, isophane, long-acting) was purchased from Novo Nordisk (Bagsværd, Denmark), at a concentration of 100 IU/mL. Excipients were zinc chloride, metacresol, glycerol, phenol, sodium hydroxide, disodium phosphate dihydrate, protamine sulphate, hydrochloric acid, and water for injection. Chitosan was from Sigma Aldrich (medium molecular weight, deacetylation: 75–85%; viscosity approximately 200–800 cP). Methylcellulose was sourced from Fluka Chemie Gmbh, USA. Hydroxyethylcellulose was purchased from Glentham Life Sciences, UK. Hydroxypropylmethylcellulose was sourced from Sigma Chemical Co., St. Louis, MO, USA. PBS (phosphate-buffered saline; pH = 7.4) was from Sigma-Aldrich, St. Louis, MO, USA. Acetic acid was from Avantor Performance Materials Poland SA, Gliwice, Poland. The reagents used were analytical grade. The Strat-M^®^ membrane was purchased from Merck Millipore (Burlington, MA, USA).

### 2.2. Preparation of Hydrogels

Preparation of the chitosan (2% *w*/*w*)–methylcellulose (4% *w*/*w*) hydrogel

First, 1 g of chitosan was dissolved in 25 g of 0.1 M acetic acid heated to 50 °C. Then, 2 g methylcellulose was dissolved in 24.5 g water with 0.5 g glycerol and heated to 80 °C. The two formulations were combined and mechanically stirred at 1000 rpm (Fisherbrand Isotemp stirring hotplate; Thermo Fisher Scientific, Mississauga, ON, Canada) until a uniform hydrogel consistency was obtained. The hydrogel matrix (CS/MC) was stored in the refrigerator at 4 °C.

Preparation of the chitosan (4% *w*/*w*)–hydroxyethylcellulose (2% *w*/*w*) hydrogel

For this purpose, 2 g of chitosan was dissolved in 25 g of 0.1 M acetic acid heated to 50 °C. Then, 1 g of hydroxyethylcellulose was dissolved in 24.5 g of water with 0.5 g of glycerol, at room temperature. The two formulations were combined and mechanically stirred at 1000 rpm (Fisherbrand Isotemp stirring hotplate; Thermo Fisher Scientific, Mississauga, ON, Canada) until a uniform hydrogel consistency was obtained. The hydrogel matrix (CS/HEC) was stored in the refrigerator at 4 °C.

Preparation of the chitosan (2% *w*/*w*)–hydroxypropylmethylcellulose (4% *w*/*w*) hydrogel

In this procedure, 1 g of chitosan was dissolved in 25 g of 0.1 M acetic acid heated to 50 °C, and 2 g of hydroxypropylmethylcellulose was dissolved in 24.5 g of water with 0.5 g of glycerol and then heated to 80 °C. The two formulations were combined and mechanically stirred at 1000 rpm (Fisherbrand Isotemp stirring hotplate; Thermo Fisher Scientific, Mississauga, ON, Canada) until a uniform hydrogel consistency was obtained. The hydrogel matrix (CS/HPMC) was stored in the refrigerator at 4 °C.

Preparation of hydrogel loaded with insulin

Then, 48 h after obtaining CS/MC, CS/HEC, and CS/HPMC hydrogels, insulin was introduced at a dose of 1 mg/g (28.57 IU/g) and mixed mechanically until transparent formulations were obtained. The obtained formulations were stored for one day at room temperature and rheological, texture, and pharmaceutical availability tests were then performed.

### 2.3. Materials Characterization

#### 2.3.1. In Vitro Pharmaceutical Availability Study

The pharmaceutical availability of insulin from the hydrogels was tested in an Erweka DT600 paddle apparatus (Husenstamm, Germany) using a Dissolution Enhancer Cell™ (exposure area of 3.80 cm^2^; Erweka, Husenstamm, Germany). Dissolution chambers were filled with 1 g INS pharmaceutical formulation and covered with Strat-M^®^ membrane (mimicking the skin barrier) according to the manufacturer’s instructions, then placed in a 200 mL vessel. The volume of PBS acceptor fluid was 50 mL. The test temperature was set at 32 ± 1 °C (temperature at the surface of human skin). The speed of rotation of the mini-paddles was 100 rpm. Sink conditions were maintained during the analysis. The amount of insulin released was analyzed by spectrophotometry at λ = 271 nm [22,23,40]. A CECIL UV-VIS spectrophotometer (CE 3021, Cambridge, UK) was used. The rectilinear dependence of absorbance on concentration was described by the equation y = 0.453x + 0.0072 (R^2^ = 0.999). The determined parameters’ mean values and the confidence intervals, standard deviation, relative standard deviation, and coefficient of variation of the obtained results indicated the adequate accuracy and precision of the method.

#### 2.3.2. Comparison of Release Profiles

The release profiles of INS from the tested pharmaceutical formulations were analyzed using statistical methods recommended by the US Food and Drug Administration (FDA) and the European Medicines Agency (EMA). A difference factor f1 of less than 15 and a similarity factor f2 greater than 50 were used as acceptance criteria for the similarity of release profiles [41]. The analysis was performed using DDSolver 1.0 software (an add-on for Microsoft Excel 2019) [42].

#### 2.3.3. Analysis of Release Kinetics

The release kinetics of INS from the developed pharmaceutical formulations were analyzed using DDSolver 1.0 software (add-on for Microsoft Excel 2019) [42]. The mathematical models used to describe the mechanism of INS release from the developed hydrogel carriers were the zero-order model, first-order model, Higuchi model, Korsmeyer–Peppas model, Peppas–Sahlin model, Hixson–Crowell model, Hopfenberg model, and Baker–Lonsdale model (Table 1). The R^2^ coefficient of determination (the higher the R^2^ value, the better the model fit), the Akaike information criterion (the lower the AIC value, the better the model fit), and the model selection criteria (the highest MSC value indicates a better model fit) were used to assess the fit of the models to the data obtained.

#### 2.3.4. Analysis of Rheological Parameters

##### Rotational Test

Characterization of the rheological parameters of the insulin hydrogels was performed with an RM 200 rotational rheometer (Lamy Rheology Instruments, Champagne au Mont d’Or, France), using the MK-CP 2445 measuring system (plate/plate with a diameter of 24 mm, angle: 0.45°). Measurement accuracy was ±1%, while repeatability was ±0.2%. The tests were conducted at 25 ± 0.1 °C (storage and retrieval temperature of the insulin hydrogel from the unit pack). A Lamy Rheology CP-1 PLUS heating system was used to maintain the set temperature during the measurements. A sample of approximately 1 mL was introduced into the measurement system and allowed to equilibrate for 30 min. After this time, the dependence of dynamic viscosity on shear rate was determined in the range 5.0–100.0 s^−1^. Measurements were taken over a period of 15 min. Thixotropy analysis was carried out using the hysteresis loop method. The specimens were subjected to shear changes with gradually increasing and then decreasing velocity. The trapezoidal method was used to determine the hysteresis loop area. The analysis was carried out using Rheomatic-P software (Version: 2.1.0.4). The relationship between shear stress and shear rate was analyzed based on selected mathematical rheological models (Table 2): Casson, Bingham, Herschel–Bulkley, Ostwald–de Waele [22,23]. The degree of fit of the model to the data was verified by the coefficient of determination R^2^, according to the assumption that the higher its value, the better the fit to the model.

##### Oscillation Test

The analysis was performed using a NETZSCH Kinexus Prime ultra+ oscillating rheometer (Selb, Germany) with an active hood cartridge, which provided temperature control during the measurements. Two oscillation tests were performed: the amplitude sweep test and the frequency sweep test. The amplitude sweep test was conducted under the following conditions: frequency 1 Hz, strain 0.1 to 100%. The frequency sweep test, on the other hand, was conducted with the following parameters: frequency 10 to 0.1 Hz, strain range of 1%. The measurement geometry used was PP40 (plate/plate with a diameter of 40 mm). A gap of 1 mm was selected for testing. Tests were conducted at 25 ± 0.01 °C (storage and retrieval temperature of the insulin hydrogel from the unit pack) and 32 ± 0.01 °C (temperature at the surface of human skin). G′ is the energy storage/elastic modulus, G″ is the loss/viscous modulus, and G* is the complex stiffness.

#### 2.3.5. Texture Analysis

Characterization of the texture parameters of insulin hydrogels was carried out using a Texture Analyzer TX-700 (Lamy Rheology Instruments, Champagne au Mont d’Or, France). A hemispherical probe with a diameter of 8 mm was used in this study. The first analysis was carried out in CRT (direct compression/relaxation/tension) mode. Compression was measured under the following conditions: compression speed, 0.5 mm/s; relaxation time (or time between cycles), 20 s; distance, 5.0 mm. The second measurement was performed using the TPA (tension/penetrometry) test, i.e., a double compression test, under the following conditions: compression speed, 0.5 mm/s; distance, 5.0 mm; force to start, 0.05 N. Hardness was the maximum force measured during the first (Hardness 1) and second compression cycles (Hardness 2). Adhesiveness was the force required to overcome the forces of attraction between the probe and the surface of the sample being analyzed. Cohesiveness was the work required to deform the hydrogel as the probe moved downwards. Elasticity was the ability of the hydrogel to deform immediately under an applied load and return to its previous shape when the load was removed. Relaxation determined how the polymer relieved stress at a constant strain [22,23,43,44,45,46]. The tests were conducted at 25 ± 0.1 °C. RheoTex software(Software version: 1.37.0.0) for TX-700, version TX-UK01/2019, was used to record and analyze the test results. The theoretical basis of the textural analysis was discussed in a previous article [23].

#### 2.3.6. Statistical Analysis

The data obtained represent the mean values, for which standard deviation values are given. A one-way ANOVA with Duncan’s test at a significance level of *p* < 0.05 was used to analyze the differences between the means. Statistica version 13.1 software (StatSoft, Cracow, Poland) was used for the calculations.

## 3. Results

An important factor limiting the therapeutic activity of a dermatological drug is the skin barrier. The active substance can accumulate on the skin surface, in the stratum corneum (adsorption), penetrate by passive diffusion into the epidermis (absorption), the dermis (penetration), and the area of the subcutaneous layer where blood vessels are located (resorption). The hydrogel matrices developed have the potential to increase the penetration of insulin (chitosan has the ability to modify transepithelial electrical resistance) [47] and prolong its contact with the application site.

In this study of the pharmaceutical availability of insulin from developed hydrogel carriers based on CS/MC, CS/HEC, and CS/HPMC, Strat-M^®^ membrane was used. It has been found that this can provide an alternative to human skin in studies of drug permeation through the skin (results of comparative studies with human skin are similar). It consists of several layers, including a tight top layer of the epidermis. In addition, it contains a combination of lipids similar to those found in the stratum corneum of human skin. The undoubted advantage of the Strat-M^®^ membrane is that pharmaceutical availability testing can be performed without animals [22,48].

**Figure 1:**
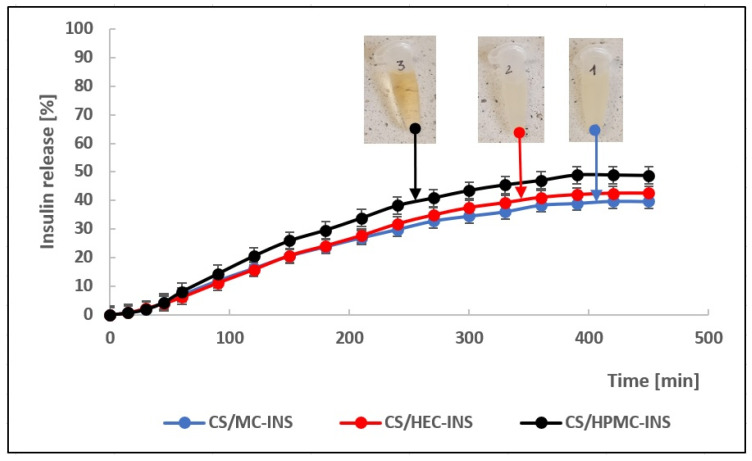
The course of insulin release profiles from chitosan / methylcellulose (CS/MC, 1), chitosan/hydroxyethylcellulose (CS/HEC, 2), chitosan/hydroxypropylmethylethylcellulose (CS/HPMC, 3). Each point corresponds to the mean ± SD value (n = 6).

Analyzing the release profiles of insulin from the developed hydrogel carriers (Figure 1), it was concluded that the hormone was released most effectively from the CS/HPMC-based hydrogel. After 6.5 h, 49% of the INS dose was released. From the other CS/HEC and CS/MC carriers, 42.5% (after 7 h) and 39.8% (after 7 h) of the insulin was released, respectively. A gradual, prolonged release of the hormone was observed. The rate of hormone release decreased over time. This was due to a decrease in API concentration as a result of erosion of the polymer matrix and prolongation of the diffusion pathway from the carrier.

The resulting release profiles were compared using the statistical methods recommended by the FDA and EMA (Table 3) [41]. The CS/HEC-INS and CS/MC-INS release profiles were found to be similar (factor f1 < 15; factor f2 > 50).

An important factor influencing the release of API from the hydrogel is its mechanism, which can be described by mathematical modeling (Table 1). Analysis of the results in Table 4 indicated that the mechanism of hormone release from the developed pharmaceutical formulations was complex (transport by diffusion and erosion) and followed the Peppas–Sahlin model. Higher values of k_PS2_ (k_PS2_/CS/MC-INS = 11.999, k_PS2_/CS/HEC-INS = 7.978, k_PS2_/CS/HPMC-INS = 18.100) compared with k_PS1_ (k_PS1_/CS/MC-INS = −20.419, k_PS1_/CS/HEC-INS = −15.056, k_PS1_/CS/HPMC-INS = −30.198) indicated that polymer relaxation and swelling affected the INS release according to non-Fickian kinetics. The “n” values calculated in the Korsmeyer–Peppas model were in the range 0.757–0.803 (0.45 < n < 0.89), confirming non-Fickian transport [49,50]. The dominant influence on the mechanism of hormone release from the developed hydrogels was polymer matrix erosion, with the minor influence of Fickian diffusion. This suggests that a loosening of the polymer chains occurred with API diffusion [51].

**Figure 2:**
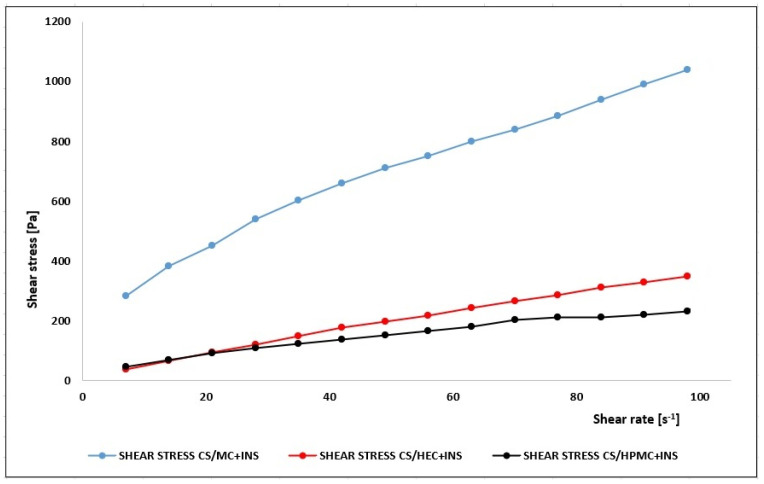
Flow rheograms of tested hydrogels at 25 °C (Lamy Rheology Instruments).

Rheological analysis of pharmaceutical preparations allows their preparation technology to be optimized in terms of the changes that may occur during storage, transport, and topical application. The rheological parameters of pharmaceutical hydrogels are an important determinant of their therapeutic effect. Based on the analysis of the rheograms (Figure 2) and the results in Table 5, it was concluded that the hydrogels studied were non-Newtonian shear-thinning fluids with yield stress. It is likely that with increasing shear rate, the structure of the polymer matrix would disintegrate and the smaller particles formed would be organized in the direction of flow, resulting in a reduction in the viscosity of the hydrogels. This characteristic promotes easier distribution of the formulation on the skin and better bioavailability of the API. This also allows the hydrogel to be efficiently filled into the unit pack and easily applied [52].

The rheograms were fitted to the selected rheological models (Table 6) and it was found that the rheological characteristics of the studied formulations were most similar to the Herschel–Bulkley model (highest values of the coefficient of determination R^2^). The values of the flow behavior indices (n = 0.596–0.93) suggest that the hydrogels can be classified as non-Newtonian pseudoplastic fluids (n < 1 indicates a non-Newtonian pseudoplastic system, n > 1 a non-Newtonian dilatant system, n = 1 a Newtonian system). The lower the value of n (CS/MC + INS: n = 0.596), the greater the shear-thinning effect and the more pronounced the pseudoplasticity [53]. The higher the n (CS/HEC + INS: n = 0.930), the lower the stability of the formulation. The yield stress determined by fitting the Herschel–Bulkley model (CS/MC + INS: τ_0_ = 90.4; CS/HEC + INS: τ_0_ = 0.050; CS/HPMC + INS: τ_0_ = 11.0) describes the minimum stress needed to maintain the hydrogel flow. The higher the value of this parameter, the stronger is the structure of the formulation. The lower the yield stress value, the better is the flowability, but the lower the retention [54]. The thixotropic properties of the hydrogels were investigated using the hysteresis loop method. The dependence of shear stress as a function of shear rate was recorded in the range of increasing shear rate from 5 to 100 s^−1^, then decreasing shear rate from 100 to 5 s^−1^. The area of the hysteresis loop, i.e., the area between the so-called forward curve and the backward curve, was calculated using the trapezoid method. The magnitude of the thixotropy field indicated the degree of hydrogel destructuring (the ability of the system to undergo different shear stresses without physicochemical modification or denaturation) [55]. The thixotropy fields were, respectively, as follows: CS/MC + INS 51712.16 Pa·s^−1^, CS/HEC + INS 16205.09 Pa·s^−1^, CS/HPMC + INS 12472.23 Pa·s^−1^. The larger the hysteresis loop field, the greater are the thixotropic properties and, consequently, the greater the stability of the formulation [56].

**Figure 3:**
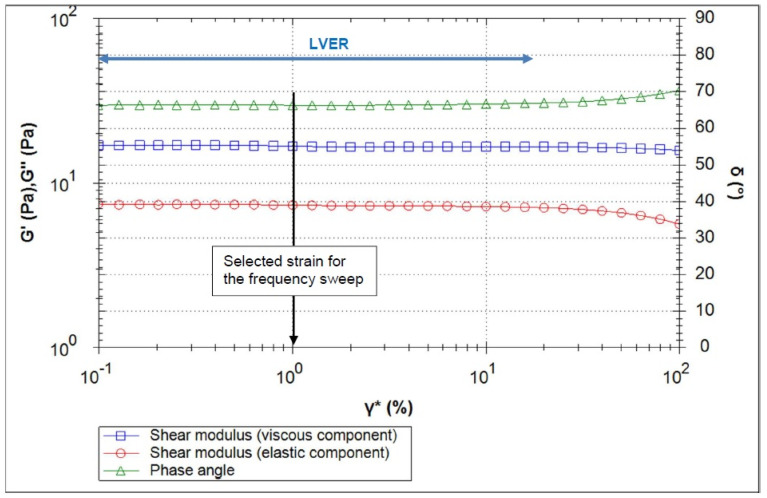
Amplitude sweep test results as a function of shear strain for CS/HEC + INS at 25 °C.

**Figure 4:**
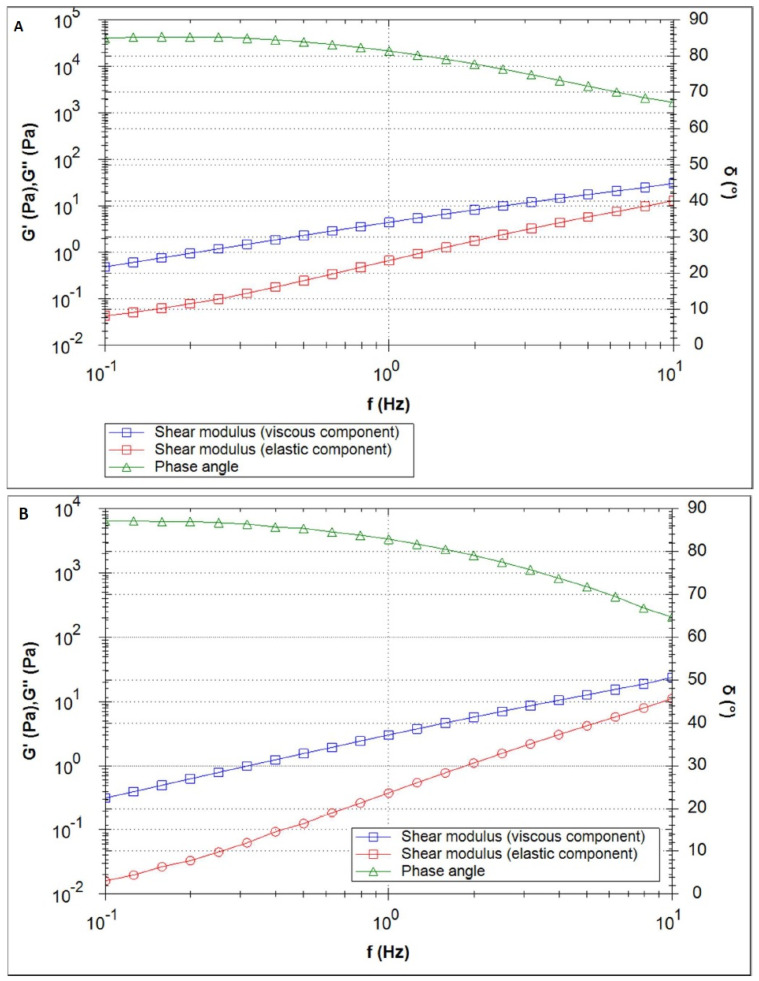
Frequency sweep of the CS/HPMC + INS sample at 25 °C (**A**) and 32 °C (**B**).

**Figure 5:**
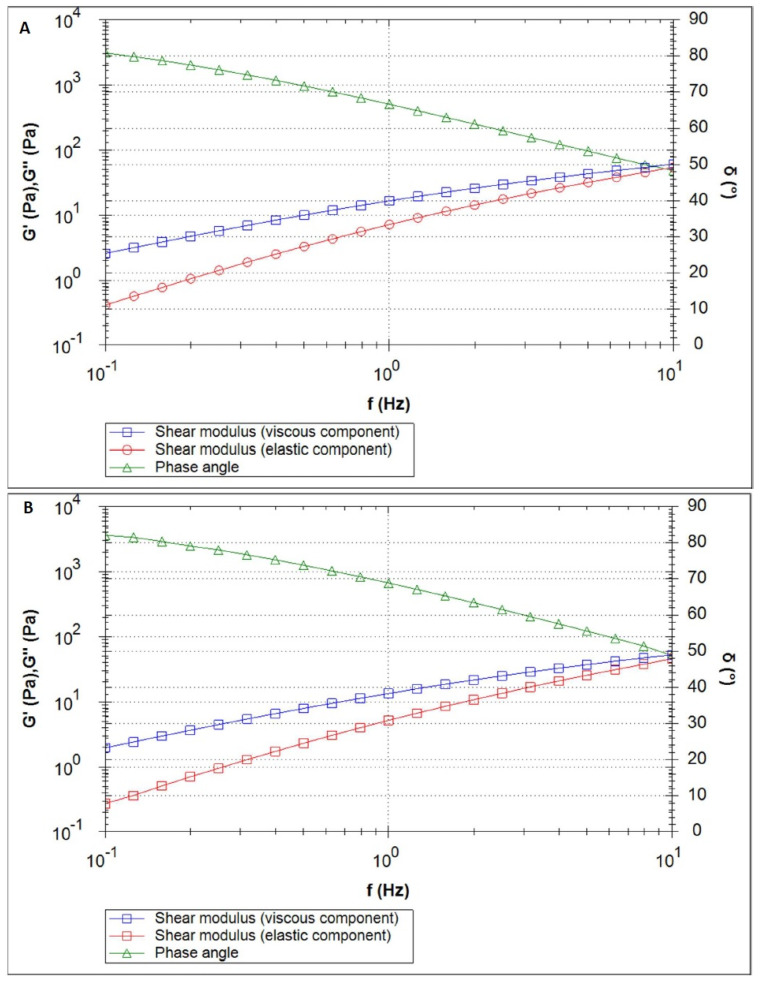
Frequency sweep of the CS/HEC + INS sample at 25 °C (**A**) and 32 °C (**B**).

**Figure 6:**
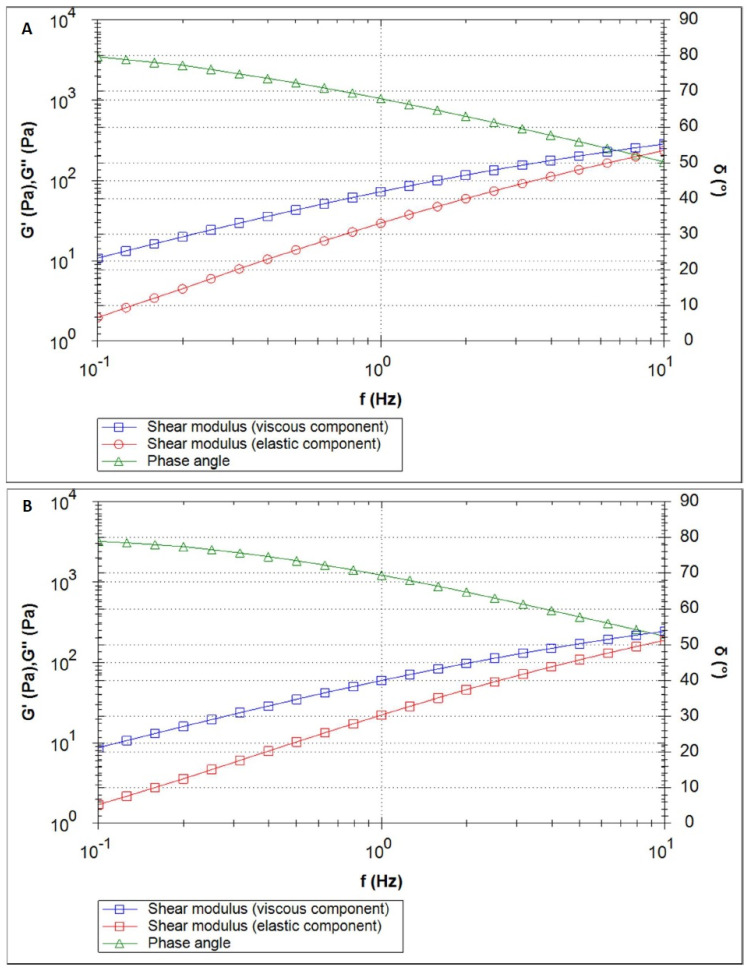
Frequency sweep of the CS/MC + INS sample at 25 °C (**A**) and 32 °C (**B**).

**Figure 7:**
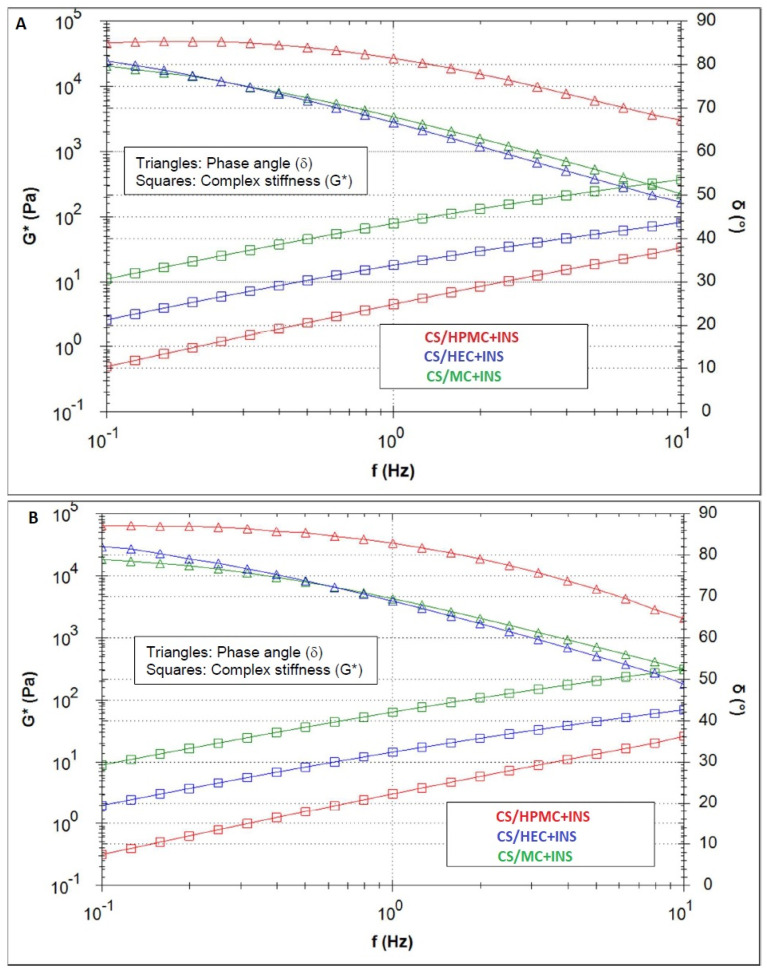
Comparison of the complex stiffness G* and the phase angle of the preparations at 25 °C (**A**) and 32 °C (**B**).

Figure 3, Figure 4, Figure 5, Figure 6 and Figure 7 show the results of the oscillatory measurements. Figure 3 shows the amplitude sweep carried out on the material CS/HEC + INS at 25 °C as an example. As long as the elastic shear modulus G′ is constant, the material is in the linear–viscoelastic range where the applied deformation does not lead to any breakdown of the sample´s structure. For the frequency sweeps, a deformation of 1% was selected.

Figure 4A depicts the curves of the elastic and viscous shear moduli (red and blue, respectively) of the sample CS/HPMC + INS along with the phase angle (green) for the measurement performed at 25 °C. In the complete frequency range, the viscous shear modulus was found to be higher than the elastic shear modulus. An increase in the temperature from 25 °C to 32 °C led to a slight decrease in the elastic shear modulus (at 1 Hz: 0.67 Pa at 25 °C vs. 0.38 Pa at 32 °C) and to an increase in the phase angle. The curves resulting from the measurement at 32 °C are displayed in Figure 4B.

Figure 5 shows the elastic and viscous moduli and the phase angle of the sample CS/HEC + INS at 25 °C and 32 °C, respectively. Here also, the viscous shear modulus was higher than the elastic shear modulus across the complete measured frequency range. As with the previous material, the temperature increase led to the expected effect: a decrease of the elastic shear modulus and an increase of the phase angle. The elastic shear modulus is about an order of magnitude higher than the previous material, while the phase angle is lower.

Figure 6 depicts the curves of the measurements carried out on the sample CS/MC + INS at 25 °C and 32 °C, respectively. The elastic shear modulus was higher than for the other materials.

The complex stiffness G* and the phase angle of the materials are compared in Figure 7A (25 °C) and Figure 7B (32 °C). For all samples, the temperature increase led to a decrease in the complex stiffness. All materials differed in their complex stiffness. For both temperatures, CS/HPMC + INS had the lowest stiffness and CS/MC + INS had the highest. The curves run parallel to each other: the lower the frequency, the lower the stiffness. In practice, this means that the slower the movement to spread the product, the “more liquid” the material feels. For both temperatures, the phase angle curves of CS/HEC + INS and CS/MC + INS were very similar, but lower than for CS/HPMC + INS, meaning a higher domination of the “liquid-like” properties for this last material.

Formulations with a higher viscous shear modulus than the elastic shear modulus show “liquid-like” behavior, where the elastic and viscous modulus increase with increasing oscillation frequency. The tanδ > 1 relationship observed in the study (tanδ = G″/G′ [57]), was also noted by other authors [57,58,59,60,61], who analyzed the effect of chitosan concentration and solvent used on the rheological and viscoelastic properties of the samples they obtained. The systems obtained showed characteristics of diluted solutions (G″ > G′) and behaved as viscoelastic liquids. It has been suggested that this may be related to the neutralization process (strong chitosan–solvent interactions form in acidic media) and the intramolecular electrostatic and steric repulsion effects exerted by the anionic residues [58]. Analyzing the viscous and elastic moduli in Figure 4, Figure 5 and Figure 6, it would be expected that an intersection of G″ and G′ will occur at a high frequency. Steffe et al. [62] suggested that polymeric materials tend to adopt a more solid form at higher frequencies.

**Figure 8:**
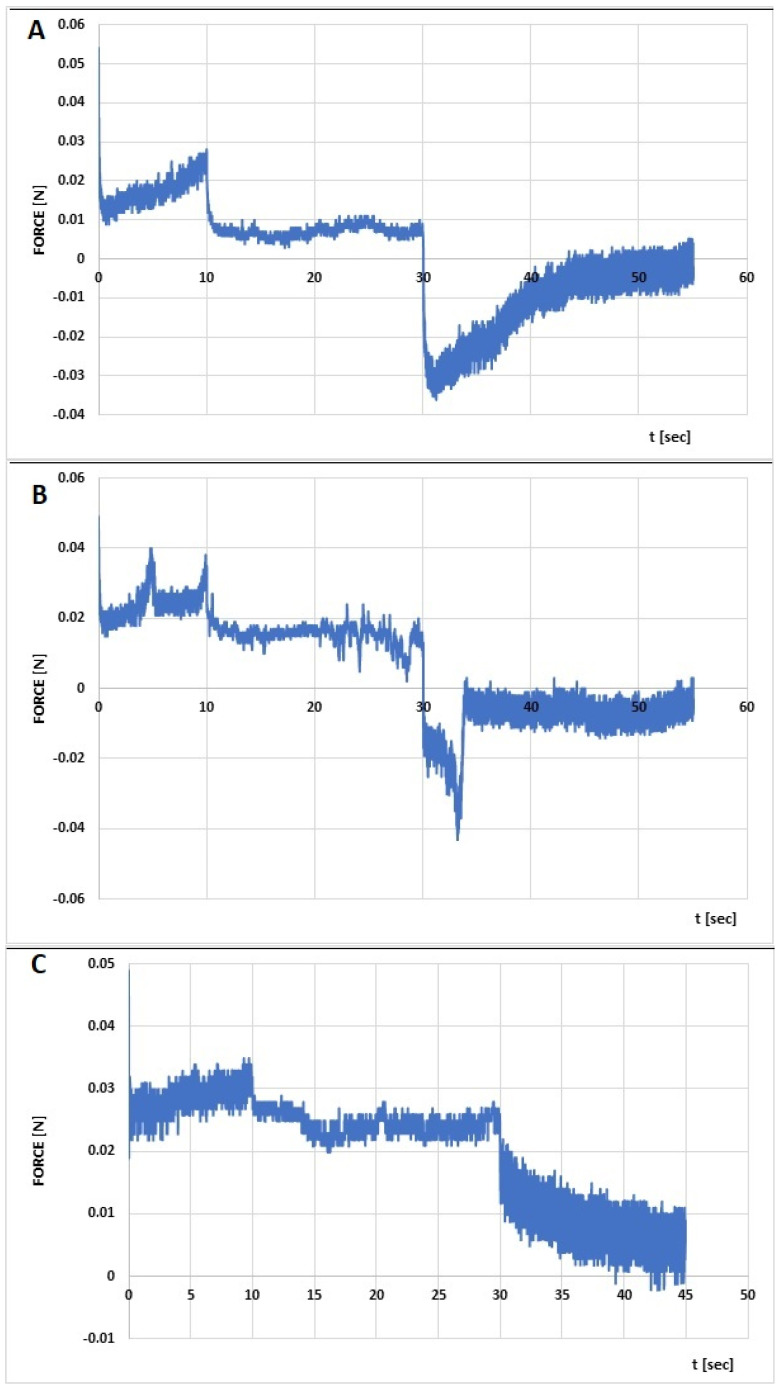
Penetration test (compression/relaxation/tension, CRT) of chitosan/methylcellulose (CS/MC) with insulin (**A**), chitosan/hydroxyethylcellulose (CS/HEC) with insulin (**B**), chitosan/hydroxypropylmethylethylcellulose (CS/HPMC) with insulin (**C**).

**Figure 9:**
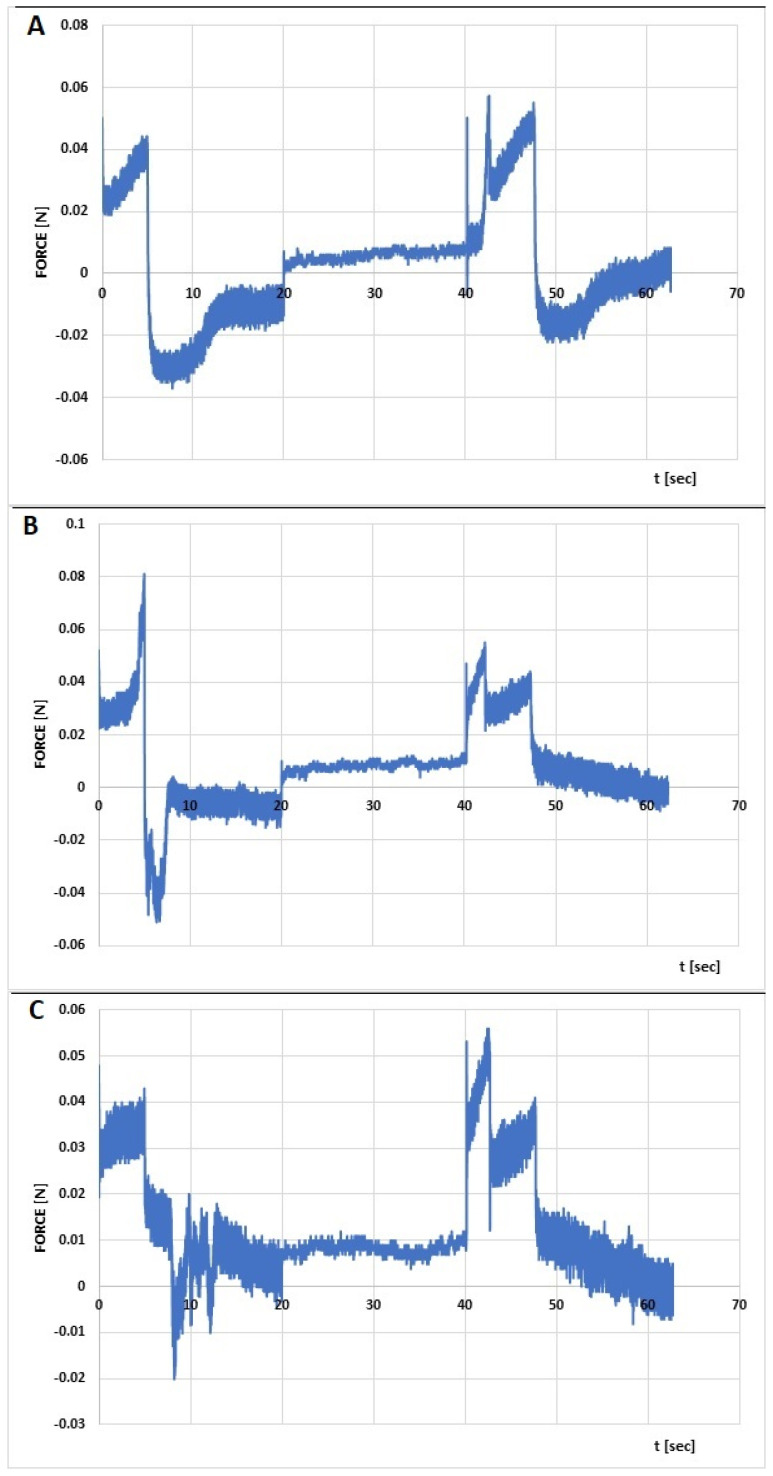
Texture profile analysis (TPA) of chitosan/methylcellulose (CS/MC) with insulin (**A**), chitosan/hydroxyethylcellulose (CS/HEC) with insulin (**B**), chitosan/ hydroxypropylmethylethylcellulose (CS/HPMC) with insulin (**C**).

The rheological characterization of the formulations was complemented by an assessment of their mechanical properties in a textural study. Relaxation, hardness 1, hardness 2, cohesiveness, adhesiveness, and elasticity were determined from the compression graphs prepared (Figure 8 and Figure 9), based on the assumptions given in a previous article [23].

The hardness of a hydrogel determines the suitability of the product for use on the skin and expresses the ease of application. The higher the value of this parameter, the harder the sample. Low values of hardness 1 for the analyzed hydrogels in the range of 0.048–0.081 N (Table 7) indicate easy spreading of the developed formulations on the skin. Smaller differences in peak heights in the first and second compression cycles (Figure 9) translate into greater flexibility of the CS/MC + INS/0.952 and CS/HPMC + INS/1.046 formulations. Cohesiveness expresses the ability of the hydrogel to structurally rebuild after application and correlates with the formulation’s performance at the application site. The higher the value of this parameter, the higher the performance of the drug. The highest values of cohesiveness were found for CS/HEC + INS and CS/MC + INS samples (1.478 and 1.373, respectively). Adhesiveness correlates with retention time at the wound site. Higher adhesiveness values ensure longer adhesion and retention of the formulation at the spread site. Potentially, the greatest adhesion to the skin surface and increased retention in the tissue environment can be provided by the CS/MC + INS hydrogel. CS/MC + INS (89.1 percent) and CS/HEC + INS (82.2 percent) showed the highest formulation resistance to deformation under gradual reduction of stress over time [23,63,64]. The obtained values of the mechanical parameters are comparable with the results obtained by other authors [65,66,67].

## 4. Discussion

Hydrogels represent a modern form of drug and exhibit many of the characteristics corresponding to an ideal dressing. A number of studies are currently underway to develop a hydrogel with the desired properties for wound healing. The optimal formulation is expected to be biocompatible, biodegradable, bioadhesive, able to conform to the shape of the wound, and to retain moisture in the wound, while allowing free gas exchange, promoting autolytic wound cleansing processes, exhibiting antimicrobial, anti-inflammatory, and antioxidant properties, and providing prolonged API release [68,69,70]. An analysis of the literature [71,72,73], on the basis of which the composition of the insulin hydrogel matrix was selected and optimized, indicates that the formulations developed show therapeutic potential in chronic wound healing.

On the basis of this study conducted on the pharmaceutical availability of insulin from the developed hydrogels through the Strat-M^®^ membrane, it was found that the release of the hormone from all formulations occurred in a prolonged manner. The lowest percentage of insulin release was observed from a chitosan-based matrix with methylcellulose. This polymer system had the highest viscosity (CS/MC + INS η (30 s^−1^) = 14.0 Pa·s) compared with the other hydrogels (CS/HEC + INS η (30 s^−1^) = 5.81 Pa·s and CS/HPMC + INS η (30 s^−1^) = 4.23 Pa·s), suggesting stronger binding between the API–carrier matrix system. The low ‘n’ values (in the Herschel–Bulkley model) for CS/MC + INS/n = 0.596 and CS/HPMC + INS/n = 0.694 indicate more structured gels. These formulations had a more elastic character. For CS/HEC + INS, a value of n = 0.930 indicates a pseudoplastic fluid with a weaker structure [74]. In general, the dose of insulin released decreased in the order CS/HPMC + INS (49%) > CS/HEC + INS (42.5%) > CS/MC + INS (39.8%), which was related not only to the viscosity of the gels but also to the surface area of the hysteresis loops obtained in the rheological study (CS/MC + INS 51712.16 Pa·s^−1^, CS/HEC + INS 16205.09 Pa·s^−1^, CS/HPMC + INS 12472.23 Pa·s^−1^, respectively). The effect of the low viscosity of the hydrogel and the small surface area of the hysteresis loop on the higher pharmaceutical availability of the API from the carrier has also been confirmed by other authors [63,75,76]. A wide hysteresis loop indicates high viscosity of the test material. A viscous matrix hinders the release of API. The higher the viscosity, the more effective is the retention of the drug in the carrier.

Characterization of the therapeutic efficacy of hydrogels is complemented by analysis of their rheological and textural parameters, allowing us to predict such properties of the formulation as spreadability on the skin, bioadhesion capacity, viscosity, and the possibility of easy application by the patient. The developed formulations exhibit non-Newtonian and pseudoplastic properties (a decrease in formulation viscosity with increasing shear stress was observed), a desirable characteristic in semi-solid drug formulations. At high shear rates, corresponding to taking the formulation directly from the unit pack, the hydrogel flows easily, spreading slightly on the tissue. In contrast, at low shear rates, the spread formulation regains its original rheological characteristics [53]. Oscillation tests, on the other hand, indicated that the hydrogels tested had a more fluid consistency (“viscoelastic liquids”) at 25 °C and 32 °C, forming a weak network of polymer chains. The trend of G′ < G″ observed in the tested systems suggests weak hydrophobic interactions (carbohydrate-carbohydrate system) between the polymer chains, forming an extended spatial structure of the hydrogel, and a predominantly viscous response [72,74,77], with a higher domination of the “liquid-like” characteristic found for the CS/HPMC + INS sample. Formulations based on cellulose derivatives with chitosan and glycerol show a desirable tendency to lower the phase transition temperature [72,73,74]. Dos Santos Carvalho et al. [74], in a study of oscillatory shear behavior in the temperature range 5–40 °C, observed similar viscous over elastic behavior for CS/MC and CS/HPMC hydrogels. This relationship changed above 40 °C (G′ > G″). On the other hand, Zanchetta et al. [72], in their oscillatory study, found an advantage for storage modulus over loss modulus in the CS/HPMC/INS system at 37 °C. This suggests that the resulting hydrogels can fill the wound surface without compromising their molecular structure and their mechanical properties [78].

Our preformulation studies indicate that the proposed hydrogel matrices can be effective carriers of insulin. The chitosan and methylcellulose-based hydrogel showed the most favorable physicochemical properties, being more viscous (its dynamic viscosity was 14.0 Pa·s at a shear rate of 30 s^−1^) and having the highest stiffness in the oscillation test compared with the other formulations. Textural analysis also showed it to have potentially the highest adhesion to the skin surface and thus increased retention in the tissue environment. Insulin was released from CS/MC/INS in a prolonged manner (39.8 percent at 7 h), ensuring effective penetration of the formulation through the stratum corneum of the skin (especially at night, when the formulation is in contact with the skin for a longer period of time). Binder et al. [79] conducted release studies of sulfadiazine sodium (SDZ, a model substance) from HPMC and HEC-based hydrogels with different concentrations and viscosities. Penetration of SDZ through the skin in vitro was analyzed using the tape-stripping method. The authors found that the depth of API penetration into the skin decreased slightly with increasing hydrogel viscosity, with the total amount of drug absorbed being independent of the matrix viscosity. The authors suggest that when developing cellulose ether-based hydrogels, moderate viscosity should be considered sufficient, allowing them to be conveniently applied by the patient. The CS/MC/INS-based “soft hydrogel” we have developed has rheological and textural properties that allow it to be conveniently applied, with the ability to be administered to wounds with irregular contours and located in hard-to-reach areas. A sterile secondary (cover) dressing may be used to keep the formulation in place and provide complete wound protection. Other authors consider “free-flowing hydrogel” to be a practical solution for treating difficult wounds [80,81]. It is also not insignificant that a finished human insulin preparation containing, among other things, metacresol and phenol with antimicrobial activity, was used to develop the INS carrier. These substances protect the hydrogel from microbial contamination in aqueous environments favorable for the growth of microorganisms, which, passing from the skin surface, can be encapsulated in the hydrogel matrix.

To date, preclinical animal and clinical studies indicate that topical application of insulin preparations is effective in wound healing, with no documented side effects [82,83,84,85]. Studies in an in vitro model of the HaCaT line suggest that the CS/HPMC/INS hydrogel can stimulate the proliferation and migration of human keratinocytes [72]. It has also been found to accelerate reepithelialization and wound gap closure under in vivo conditions in a cutaneous wound model by interacting with its receptor [9,72]. Dermatological preparations of insulin have been confirmed to have no effect on blood glucose levels [72,86,87]. Importantly, the insulin molecule incorporated into hydrogel systems remains stable and bioactive [23,72,88].

## 5. Conclusions

Dermatological insulin hydrogels have been developed for preparation in a pharmacy formulation setting. The CS/MC/INS pharmaceutical formulation showed an acceptable balance between rheological and textural parameters and ease of application. The API was released from the carrier in a prolonged manner, eliminating the need to apply the formulation several times per day. We suggest further preclinical and clinical studies to confirm the therapeutic efficacy of insulin hybrid hydrogels in chronic wound healing.

## Figures and Tables

**Table 1:** Characteristics of selected mathematical models used to evaluate the release of INS from hydrogels [42].

Kinetics Models	Equation	Parameters Definition
Zero-order	f = k_0_ × t	f, amount of the drug released; t, time; k_0_, reaction rate coefficient.
First-order	f = 100 × [1 − e^−k1 × t^]	f, amount of the drug released; t, time; k_1_, rate constant.
Higuchi	f = k_H_ × t^0.5^	f, amount of the drug released; t, time; k_H_, dissolution constant.
Korsmeyer–Peppas	f = k_KP_ × t^n^	f, amount of the drug released; t, time; k_KP_, constant depicting the experimental parameters based on geometry and dosage forms; n, release exponent; n ≤ 0.45 Fickian diffusion; 0.45 < n < 0.89 non-Fickian transport; n = 0.89 case II (relaxation) transport; n > 0.89 super case II transport mechanism.
Peppas–Sahlin	f = k_PS1_ × t^m^ + k_PS2_ × t^(2 × m)^	f, amount of the drug released; t, time; k_PS1_, Peppas–Sahlin release constant (constant for Fickian diffusion); k_PS2_, constant for case II relaxational mechanism; m, diffusion exponent.
Hixson–Crowell	f = 100 × [1 − (1 − k_HC_ × t)^3^]	f, amount of the drug released; t, time; k_HC_, Hixson–Crowell release constant.
Hopfenberg	f = 100 × [1 − (1 − k_HB_ × t)^n^]	f, amount of the drug released; t, time; n, release exponent; k_HB_, Hopfenberg release constant.
Baker–Lonsdale	3/2 × [1 − (1 − F/100)^2/3^] − *F*/100= k_BL_ × t	f, amount of the drug released; t, time; k_BL_, Baker–Lonsdale release constant.

**Table 2:** Characteristics of selected rheological models [22,23].

Rheological Models	Equation	Parameters Definition
Ostwald–de Waele	τ = K × $\dot{\gamma }$ ^n^	τ, shear stress [Pa]; K, consistency coefficient [Pa]^1/2^[s]^n^; $\dot{\gamma }$, shear rate [s^−1^]; n, flow behavior index.
Herschel–Bulkley	τ = τ_0_ + K × $\dot{\gamma }$^n^	τ, shear stress [Pa]; τ_0_, yield stress or yield point; K, consistency coefficient [Pa]^1/2^[s]^n^; $\dot{\gamma }$, shear rate [s^−1^]; n, flow behavior index.
Bingham	τ = τ_0_ + η × $\dot{\gamma }$	τ, shear stress [Pa]; τ_0_, yield stress or yield point; γ, viscosity [Pa·s]; $\dot{\gamma }$, shear rate [s^−1^].
Casson	τ^0.5^ = τ_0_^0.5^ + η^0.5^ × $\dot{\gamma }$^0.5^	τ, shear stress [Pa]; τ_0_, yield stress or yield point; γ, viscosity [Pa·s]; $\dot{\gamma }$, shear rate [s^−1^].

**Table 3:** Comparison of the release profiles of CS/MC, CS/HEC, and CS/HPMC.

Formula Code	f1 $f1=\left[\frac{\sum \left|R_{t}-T_{t}\right|}{\sum R_{t}}\right]\;\times \;100$	f2 $f2=50\times \log\left\{{\left[1+\frac{1}{n}\sum {\left(R_{t}-T_{t}\right)}^{2}\right]}^{-0.5}\times \;100\right\}$	Dissolution Profile
CS/MC-INS			
vs. CS/HEC-INS	6.17	84.21	Similar
CS/MC-INS			
vs. CS/HPMC-INS	24.02	58.95	Dissimilar
CS/HEC-INS			
vs. CS/HPMC-INS	18.34	64.67	Dissimilar

**Table 4:** Mathematical models describing the kinetics of insulin release from CS/MC-INS, CS/HEC-INS, and CS/HPMC-INS hydrogels.

Kinetics Models	Hydrogel	Parameters	R^2^ Adjusted	AIC	MSC
Zero-order	CS/MC-INS	k0 = 0.106	0.9398	99.6577	2.5555
	CS/HEC-INS	k0 = 0.113	0.9531	98.1674	2.8163
	CS/HPMC-INS	k0 = 0.132	0.9332	109.6961	2.4551
First-order	CS/MC-INS	k1 = 0.001	0.9768	82.5242	3.5073
	CS/HEC-INS	k1 = 0.001	0.9820	80.9338	3.7737
	CS/HPMC-INS	k1 = 0.002	0.9780	89.7122	3.5653
Higuchi	CS/MC-INS	kH = 1.849	0.9139	106.0903	2.1981
	CS/HEC-INS	kH = 1.955	0.8975	112.2405	2.0344
	CS/HPMC-INS	kH = 2.297	0.9064	115.7729	2.1175
Korsmeyer–Peppas	CS/MC-INS CS/HEC-INS CS/HPMC-INS	k_KP_ = 0.434 n = 0.757 k_KP_ = 0.352 n = 0.803 k_KP_ =0.536 n = 0.758	0.9725 0.9720 0.9650	86.4733 89.8124 98.9581	3.2879 3.2804 3.0517
Peppas–Sahlin	CS/MC-INS CS/HEC-INS CS/HPMC-INS	k_PS1_ = −20.419 k_PS2_ = 11.999 m = 0.175 k_PS1_ = −15.056 k_PS2_ = 7.978 m = 0.206 k_PS1_ = −30.198 k_PS2_ = 18.100 m = 0.165	0,9898 0.9865 0.9858	69.5147 77.4582 83.5061	4.2301 3.9668 3.9101
Hixson–Crowell	CS/MC-INS CS/HEC-INS CS/HPMC-INS	k_HC_ = 0.000 k_HC_ = 0.000 k_HC_ = 0.001	0.9678 0.9761 0.9689	88.3766 86.0249 95.9302	3.1822 3.4909 3.2199
Hopfenberg	CS/MC-INS CS/HEC-INS CS/HPMC-INS	k_HB_ = 0.000 n = 275.349 k_HB_ = 0.000 n = 645.651 k_HB_ = 0.0 n = 1114.896	0.9752 0.9809 0.9766	84.5870 82.9537 91.7268	3.3927 3.6615 3.4534
Baker–Lonsdale model	CS/MC-INS	kBL = 0.0	0.8975	109.2393	2.0232
	CS/HEC-INS	kBL = 0.0	0.8780	115.3791	1.8601
	CS/HPMC-INS	kBL = 0.0	0.8847	119.5170	1.9095

**Table 5:** Viscosity values at different shear rates (mean ± SD, n = 3, T = 25 ± 0.1 °C).

Hydrogel	η (30 s^−1^) [Pa·s]	η (50 s^−1^) [Pa·s]	η (100 s^−1^) [Pa·s]
CS/MC + INS	14.0 ± 0.201	8.08 ± 0.423	5.84 ± 0.467
CS/HEC + INS	5.81 ± 0.343	3.22 ± 0.190	2.68 ± 0.201
CS/HPMC + INS	4.23 ± 0.131	2.94 ± 0.153	2.12 ± 0.303

**Table 6:** The results obtained from mathematical modeling of the rheograms.

Hydrogel	Herschel–Bulkley				Ostwald–de Waele			Bingham		Casson	
	τ_0_	N	K	R^2^	n	K	R^2^	τ_0_	R^2^	τ_0_	R^2^
CS/MC + INS	90.40	0.596	60.90	0.998	0.495	104.70	0.996	300.2	0.985	145.3	0.995
CS/HEC + INS	0.05	0.930	4.98	0.999	0.930	4.98	0.999	12.3	0.998	0.792	0.999
CS/HPMC + INS	11.00	0.694	9.60	0.997	0.621	13.90	0.996	47.8	0.979	17.0	0.991

Symbols: τ_0_, yield stress [Pa]; K, consistency index [Pa*s^n^]; n, flow behavior index; R^2^, regression coefficient.

**Table 7:** Mechanical parameters of hydrogels (mean ± SD, n = 3, T = 25 ± 0.1 °C).

Formula Code	Relaxation [%]	Hardness 1 [N]	Hardness 2 [N]	Cohesiveness	Adhesiveness [mJ]	Elasticity
CS/MC + INS	89.1	0.050	0.057	1.373	0.3	0.952
CS/HEC + INS	82.2	0.081	0.055	1.478	0.2	0.747
CS/HPMC + INS	49.4	0.048	0.056	1.000	0.1	1.046
*p*	<0.05	<0.05	NS	<0.05	<0.05	<0.05

## Data Availability

The data presented in this study are available upon request from the corresponding authors.
